# Lipid profile and future risk of exudative age-related macular degeneration development: a nationwide cohort study from South Korea

**DOI:** 10.1038/s41598-022-23607-w

**Published:** 2022-11-05

**Authors:** Sungsoon Hwang, Se Woong Kang, Jaehwan Choi, Ki Young Son, Dong Hui Lim, Dong Wook Shin, Kyunga Kim, Sang Jin Kim

**Affiliations:** 1 Department of Ophthalmology, Samsung Medical Center, Sungkyunkwan University School of Medicine, #81 Irwon-Ro, Gangnam-Gu, Seoul, 06351 Republic of Korea; 2 Department of Clinical Research Design and Evaluation, Samsung Advanced Institute for Health Sciences and Technology (SAIHST), Sungkyunkwan University, Seoul, Republic of Korea; 3 Department of Family Medicine and Supportive Care Center, Samsung Medical Center, Sungkyunkwan University School of Medicine, Seoul, Republic of Korea; 4 Department of Digital Health, Samsung Advanced Institute for Health Sciences and Technology (SAIHST), Sungkyunkwan University, Seoul, Republic of Korea; 5 Statistics and Data Center, Research Institute for Future Medicine, Samsung Medical Center, Seoul, Republic of Korea

**Keywords:** Macular degeneration, Epidemiology

## Abstract

This nationwide population-based cohort study evaluated the association between lipid profiles and the future risk of exudative age-related macular degeneration (AMD) using authorized clinical data provided by the Korean National Health Insurance Service. A total of 6,129,616 subjects over 50 years of age who participated in the Korean National Health Screening Program in 2013 or 2014 were included. Data on risk factors, including age, sex, comorbidities, behavioral factors, and baseline lipid profiles, including total cholesterol, high-density lipoprotein (HDL) cholesterol, low-density lipoprotein (LDL) cholesterol, and triglyceride (TG) levels were collected. Patients were followed-up patients until December 2018, and incident cases of exudative AMD were identified using registered diagnostic codes. During an average follow-up period of 4.91 years, 18,803 patients were newly diagnosed with exudative AMD. Compared to the lowest HDL cholesterol quartile group, the highest HDL cholesterol quartile group had a greater risk of future exudative AMD development with a hazard ratio (95% confidence interval) of 1.13 (1.08–1.18) in the fully adjusted model. The highest TG quartile group had a lower risk of exudative AMD than the lowest TG quartile group, with a hazard ratio (95% confidence interval) of 0.84 (0.81–0.88). High HDL cholesterol and low TG levels were prospectively associated with exudative AMD incidence.

## Introduction

Age-related macular degeneration (AMD) is a leading cause of vision loss among the elderly^1^, with a prevalence of 8.69% among individuals over 45 years old worldwide. The estimated number of patients with AMD worldwide in 2020 was 196 million, indicating a substantial healthcare-related burden^2^. AMD is etiologically complex and has many systemic, environmental, and behavioral risk factors^3,4^. Classically, cardiovascular risk factors, such as hypertension, diabetes, dyslipidemia, and smoking, have been reported to be linked to AMD^5,6^. Recent advancements in genetics have revealed genetic variants associated with AMD and provided clues for understanding the pathophysiologic process involved in AMD^7^. These identified genetic variants were relevant to various domains including complement activity, lipid metabolism, extracellular matrix remodeling, and angiogenesis^8–10^.

Drusen is a hallmark of AMD, and lipid compose more than 40% of drusen^11^. Therefore, lipid metabolism has long been believed to be associated with the pathogenesis of AMD. Previous classic observational studies showed conflicting results and did not attain a consensus on how each lipid component affects the development of AMD^12–14^. In the past decade, genome-wide association studies have revealed that some AMD-associated genetic variants are closely related to genes encoding the components of lipid metabolism pathways^7^. More recently, Mendelian randomization studies revealed that genetically determined high-density lipoprotein (HDL) cholesterol levels are associated with AMD^15–17^.

Although Mendelian randomization studies have provided strong evidence of causation, these previous studies had the following limitations. First, they mainly observed patients of European descent; thus, their results cannot be generalized to other ethnicities. Second, the results regarding the association of low-density lipoprotein (LDL) cholesterol and triglycerides (TG) with the risk of AMD were inconsistent. Third, the number of subjects included in prior studies was small. These drawbacks necessitate large-scale real-world evidence for discerning the association between lipid profiles and AMD in populations of various ethnicity. Therefore, we investigated the prospective association of lipid profiles with the risk of incident exudative AMD using a large and nationally representative insurance dataset from South Korea.

## Methods

### Setting

This was a nationwide, population-based, retrospective cohort study using data provided by the Korea National Health Insurance Service (NHIS). The study adhered to the tenets of the Declaration of Helsinki and was approved by the Institutional Review Board of Samsung Medical Center, Seoul, Republic of Korea (IRB File Number 2020-02-126). The board waived the requirement for informed consent based on the use of de-identified public data and the retrospective study design.

The NHIS provides mandatory universal medical care for all registered citizens in South Korea. The NHIS holds medical information for the entire population, including demographic information, mortality data, and health claims data. Demographic data included age, sex, and income, and the claims data included the date of clinical visits, prescription records, and diagnostic codes defined by the Korean Classification of Diseases 7th revision (KCD-7), which is based on the International Classification of Diseases, 10th revision, but with a few changes specific to Korea. The NHIS also contains data from the National Health Screening Program (NHSP). The NHSP is a free biennial general health examination offered to all Koreans aged over 40 years, provided by the NHIS. The data from the NHSP included patients’ responses to structured questionnaires, anthropometric measurements, and laboratory test results. Demographic, mortality, health claims, and health screening data can be linked together through deidentified key numbers assigned to each individual. This database has been widely used in previous studies to identify associations between various diseases and risk factors. The detailed database profile information has been provided elsewhere^18^.

### Definition of exudative age-related macular degeneration

Since August 2009, exudative AMD has been registered as an intractable rare disease in the Korean Health Insurance system. All patients diagnosed with exudative AMD are given a special registration code (V201) in the NHIS database and are registered in the “copayment deduction program,” which pays back 90% of medical expenses related to exudative AMD, including costs for imaging and intraocular anti-VEGF injections. To be registered in this program, a board-certified ophthalmologist must provide medical records, fundus photography, optical coherence tomography, and fluorescein angiography to support the diagnosis of exudative AMD for the Health Insurance Review and Assessment Service, and the diagnosis has to be re-confirmed by other ophthalmologists. Therefore, all cases of multimodal imaging-confirmed exudative AMD can be identified using the registration code V201 in the NHIS database. Detailed descriptions of the copayment deduction for exudative AMD are available elsewhere^19^.

### Subjects

**Figure 1:**
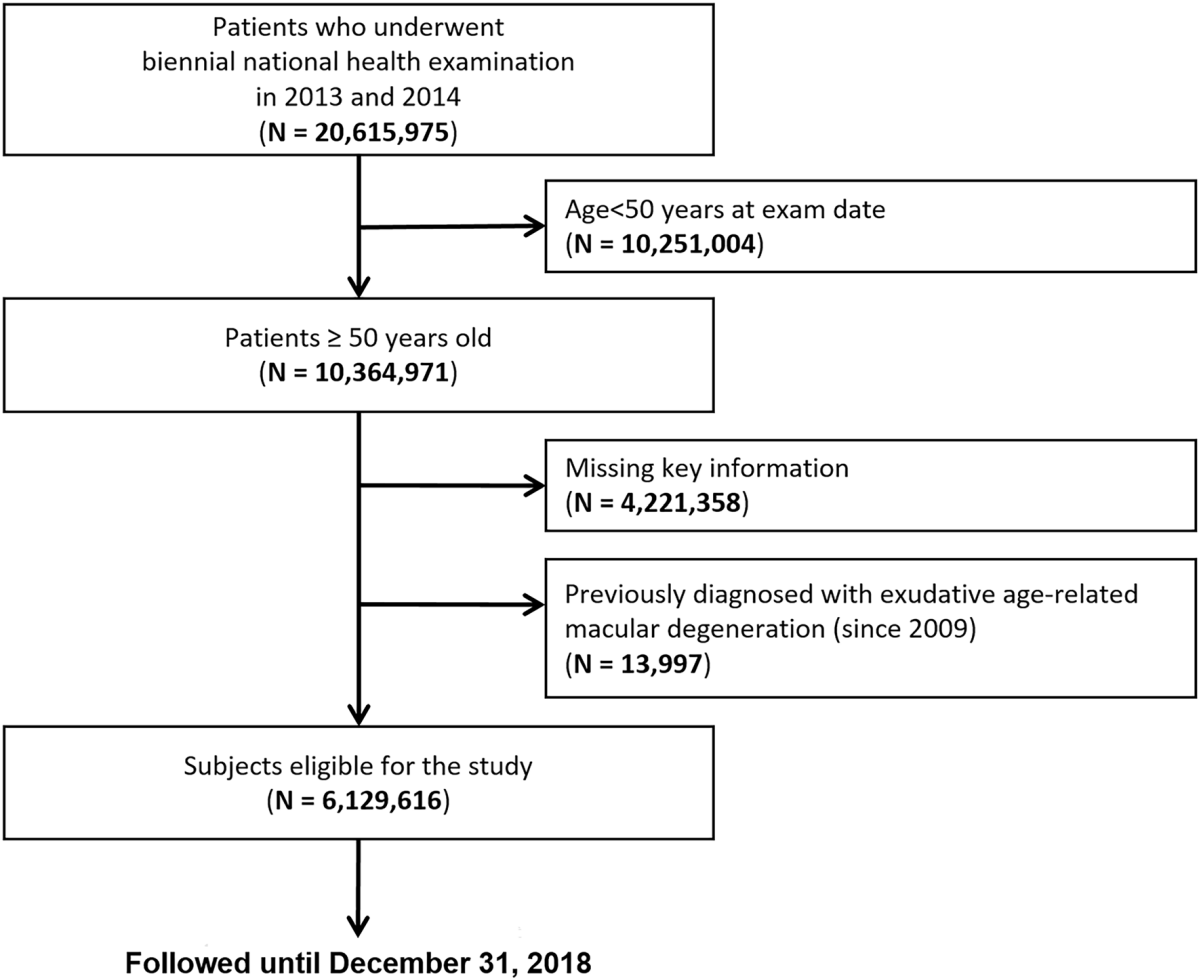
Flow chart of the cohort study design.

Among 10,364, 971 subjects over 50 years of age who underwent the National Health Screening examination in 2013 and 2014, we identified a total of 6,143,613 eligible participants without missing key information relevant for analyses. We excluded individuals who had been diagnosed with exudative AMD (special registration code V201) prior to the examination date (n = 13,997) because the study outcome was incident cases of exudative AMD that developed after the examination. Finally, 6,129,616 subjects over 50 years of age without a history of exudative AMD were included in the study (Fig. 1).

### Exposures and covariates

For the definition of exposures and covariates, the methodology of a previously published nationwide epidemiologic study was used^20^.

The total cholesterol, HDL cholesterol, LDL cholesterol, and TG, together with glucose and creatinine were measured from blood samples collected after overnight fasting. Total cholesterol level, HDL cholesterol level, LDL cholesterol level, and TG level were categorized into four groups using cut-off values of lower quartile, median and upper quartile.

Possible confounding factors were comprehensively assessed. We identified the use of dyslipidemia medication based on patients’ responses to the health screening questionnaire and prescription codes (corresponding to ATC code C10) for lipid-lowering medication within a year before the health screening examination. Comorbid hypertension and diabetes were identified based on self-reported questionnaire responses, health screening measurement results regarding blood pressure (hypertension, systolic blood pressure ≥ 140 mmHg or diastolic blood pressure ≥ 90 mmHg) and fasting glucose (diabetes, fasting blood glucose levels ≥ 126 mg/dL), and presence of diagnostic codes (KCD-7 code: I15 for hypertension, E11–E14 for diabetes) combined with medication prescription codes within a year prior to the health screening examination. Stroke and heart diseases were identified based on the patient-reported health screening questionnaire. Chronic kidney disease was defined as having an estimated glomerular filtration rate of < 60 ml/min/1.73m^2^ calculated from serum creatinine level. Income level was categorized into quartiles according to the insurance premium level, which was determined by the total household income. We also collected data regarding health-related behaviors based on the participants’ responses to the health screening questionnaire. Smoking status was divided into none, past, and current. Drinking habit was categorized into none, mild (< 6 times a week), and heavy (≥ 6 times a week). Regular exercise was defined as performing a moderate level of physical activity for more than 30 min a day for more than 5 days per week. The body mass index (BMI) was calculated as the weight (kg) divided by height squared (m^2^), and categorized as underweight (BMI < 18.5 kg/m^2^), normal weight (18.5 ≤ BMI < 23 kg/m^2^), overweight (23 ≤ BMI < 25 kg/m^2^), obese I (25 ≤ BMI < 30 kg/m^2^), and obese II (≥ 30 kg/m^2^), according to the Korean Society for the Study of Obesity^21^.

### Outcome and follow-up

We regarded individuals with registration code V201 after the baseline examination as incident cases of exudative AMD. Moreover, for each case, we defined the first date of V201 registration as the time of exudative AMD incidence. We followed patients from the date of the health check-up to the date of exudative AMD incidence, death, or the end of the study period (December 31, 2018), whichever came first.

### Statistical analyses

We calculated the incidence rates for exudative AMD by dividing the number of incident cases by the total number of person-years. We calculated hazard ratios (HRs) and 95% confidence intervals (CIs) using Cox proportional hazard models. The variables adjusted for each Cox model were as follows: model 1, age and sex; model 2, demographic factors (age, sex, and income level) and systemic comorbidities (hypertension, diabetes, stroke, heart diseases, chronic kidney disease, and use of dyslipidemia medication at baseline); and model 3, demographic factors, systemic comorbidities, and behavioral factors (smoking status, drinking habit, regular exercise, and BMI). We also conducted sensitivity analyses excluding subjects who were taking lipid-lowering medication at the time of baseline examination because taking lipid-lowering medication could largely influence the lipid profile of individuals. All statistical analyses were performed using SAS version 9.4 (SAS Institute Inc., Cary, NC, USA). P-values were two-sided and considered statistically significant at values less than 0.05.

## Results

### Baseline characteristics

Table 1 shows the detailed baseline characteristics of the study participants. The average age of the study participants was 60.80 years. The lower quartile, median, and upper quartile of lipid profile were 172, 196 and 222 mg/dL for total cholesterol, 44, 52 and 62 mg/dL for HDL cholesterol, 94, 117 and 140 mg/dL for LDL cholesterol, and 79, 112 and 161 mg/dL for TG. The average follow-up period was 4.91 years and 18,803 patients were newly diagnosed with exudative AMD during the follow-up period. Supplementary Table 1 demonstrates the baseline parameters of the study subjects according to the presence of exudative AMD during the study period.

**Table 1:** Baseline characteristics of the study population.

Variables	Total (N = 6,129,616)
**1. Demographic Factors**	
Age, years, mean ± SD	60.80 ± 8.43
Age group, No. (%)	
50–59 years	3,148,558 (51.37)
60–69 years	1,849,161 (30.17)
70–79 years	956,891 (15.61)
80–89 years	165,953 (2.71)
≥ 90 years	9,053 (0.15)
Sex, No. (%)	
Male	2,933,702 (47.86)
Female	3,195,914 (52.14)
Income, No. (%)	
Q1 (lowest)	1,278,484 (20.86)
Q2	1,093,932 (17.85)
Q3	1,418,257 (23.14)
Q4 (highest)	2,338,943 (38.16)
**2. Systemic Comorbidities**	
Hypertension, No. (%)	
No	3,581,010 (58.42)
Yes	2,548,606 (41.58)
Diabetes mellitus, No. (%)	
No	4,982,740 (81.29)
Yes	1,146,876 (18.71)
Dyslipidemia medication, No. (%)	
No	4,599,366 (75.04)
Yes	1,530,250 (24.96)
Stroke, No. (%)	
No	6,023,537 (98.27)
Yes	106,079 (1.73)
Heart diseases, No. (%)	
No	5,876,515 (95.87)
Yes	253,101 (4.13)
Chronic kidney disease, No. (%)	
No	5,707,208 (93.11)
Yes	422,408 (6.89)
**3. Behavioral Factors**	
Smoking history, No. (%)	
Never smoked	4,028,729 (65.73)
Former smoker	1,128,032 (18.40)
Current smoker	972,855 (15.87)
Drinking habit, No. (%)	
None	3,920,201 (63.96)
Mild	1,920,124 (31.33)
Heavy	289,291 (4.72)
Regular physical activity, No. (%)	
No	4,691,764 (76.54)
Yes	1,437,852 (23.46)
Body mass index, No (%)	
< 18.5 kg/m^2^	140,262 (2.29)
18.5 to < 23 kg/m^2^	2,130,907 (34.76)
23 to < 25 kg/m^2^	1,656,430 (27.02)
25 to < 30 kg/m^2^	1,986,465 (32.41)
≥ 30 kg/m^2^	215,552 (3.52)
**4. Examination results**	
Systolic blood pressure, mmHg, mean ± SD	124.58 ± 14.88
Diastolic blood pressure, mmHg, mean ± SD	76.74 ± 9.71
Fasting plasma glucose, mg/dL, mean ± SD	103.33 ± 25.83
Creatinine, mg/dL, mean ± SD	0.90 ± 0.49
Total cholesterol, mg/dL, mean ± SD	197.89 ± 38.56
Total cholesterol group, No. (%)	
Q1 (< 172 mg/dL)	1,524,313 (24.87)
Q2 (172 to < 196 mg/dL)	1,496,337 (24.41)
Q3 (196 to < 222 mg/dL)	1,549,911 (25.29)
Q4 (≥ 222 mg/dL)	1,559,055 (25.43)
HDL cholesterol, mg/dL, mean ± SD	53.83 ± 15.10
HDL cholesterol group, No. (%)	
Q1 (< 44 mg/dL)	1,450,022 (23.66)
Q2 (44 to < 52 mg/dL)	1,524,625 (24.87)
Q3 (52 to < 62 mg/dL)	1,614,880 (26.35)
Q4 (≥ 62 mg/dL)	1,540,089 (25.13)
LDL cholesterol, mg/dL, mean ± SD	118.14 ± 39.24
LDL cholesterol group, No. (%)	
Q1 (< 94 mg/dL)	1,517,736 (24.76)
Q2 (94 to < 117 mg/dL)	1,547,973 (25.25)
Q3 (117 to < 140 mg/dL)	1,493,483 (24.37)
Q4 (≥ 140 mg/dL)	1,570,424 (25.62)
TG, mg/dL, mean ± SD	133.02 ± 87.87
TG group, No. (%)	
Q1 (< 79 mg/dL)	1,503,719 (24.53)
Q2 (79 to < 112 mg/dL)	1,548,052 (25.26)
Q3 (112 to < 161 mg/dL)	1,532,448 (25.00)
Q4 (≥ 161 mg/dL)	1,545,397 (25.21)

*AMD* age-related macular degeneration, *SD* standard deviation, *Q* quartile, *HDL* high-density lipoprotein, *LDL* low-density lipoprotein, *TG* triglycerides.

### Lipid profile and exudative age-related macular degeneration

**Figure 2:**
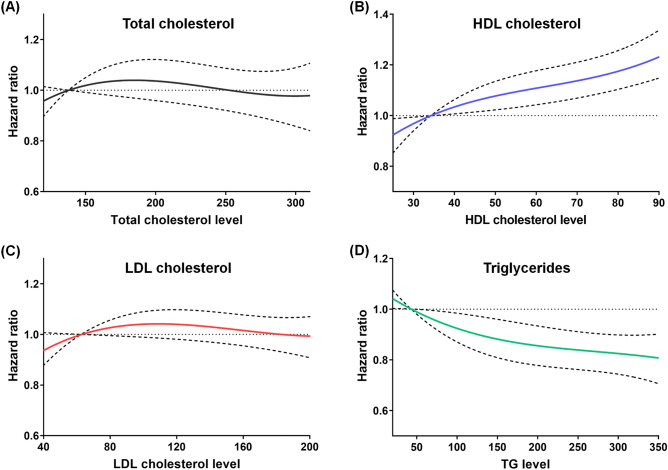
Restricted cubic spline curves presenting the adjusted hazard ratio for exudative age-related macular degeneration incidence according to total cholesterol, high-density lipoprotein cholesterol, low-density lipoprotein cholesterol, and triglyceride level. Curves represent HRs (solid lines) and their 95% CIs (dashed lines) based on restricted cubic splines for baseline total cholesterol (**A**), HDL cholesterol (**B**), LDL cholesterol (**C**), and triglyceride (**D**) levels with knots at the 5th, 35th, 65th, and 95th percentiles and the reference point at the 5th percentile of their distributions. The risk of exudative age-related macular degeneration continuously increased as HDL cholesterol levels increased (**B**) and triglyceride levels decreased (**D**) in the Cox proportional hazards model fully adjusted for demographic factors, systemic comorbidities, and behavioral factors (model 3).

Table 2 presents the incidence rates and HRs with 95% CIs of exudative AMD according to total cholesterol, HDL cholesterol, LDL cholesterol, and TG levels in the various fitting models. The incidence rate of exudative AMD was 62.76 per 100,000 person-years in the study subjects. In the fully adjusted analysis (model 3), the highest HDL cholesterol quartile group had a greater risk of exudative AMD than the lowest HDL cholesterol quartile group with an HR (95% CI) of 1.13 (1.08–1.18), and the highest TG quartile group had a lower risk of exudative AMD than the lowest TG quartile group with an HR (95% CI) of 0.84 (0.81–0.88). Figure 2 presents the restricted cubic spline curves of the adjusted HR for incident exudative AMD according to total cholesterol, HDL cholesterol, LDL cholesterol, and TG levels. The restricted cubic spline models showed a continuous increase in HR as HDL cholesterol level increased, and a continuous decrease in HR as TG level increased. Total cholesterol and LDL cholesterol levels did not significantly influence the HR for exudative AMD. Supplementary Figs. 1, 2, 3 and 4 demonstrate the subgroup analyses for prospective association between the risk of incident exudative AMD and total cholesterol (Supplementary Fig. 1), HDL cholesterol (Supplementary Fig. 2), LDL cholesterol (Supplementary Fig. 3), and TG level (Supplementary Fig. 4). The results were consistent across various subgroups.

**Table 2:** Hazard ratios and 95% confidence intervals for development of exudative age-related macular degeneration according to baseline lipid profile.

	Subject No	Case No	Duration (person-years)	IR per 100,000 person-years	Model 1	Model 2	Model 3
					HR (95% CI)	HR (95% CI)	HR (95% CI)
Overall	6,129,616	18,803	29,961,137	62.76			
**Baseline total cholesterol**							
Q1 (lowest)	1,524,313	5,970	7,352,593	81.20	1.00 (reference)	1.00 (reference)	1.00 (reference)
Q2	1,496,337	4,702	7,327,003	64.17	0.95 (0.91–0.98)	1.00 (0.96–1.04)	1.00 (0.96–1.04)
Q3	1,549,911	4,373	7,615,387	57.42	0.94 (0.90–0.97)	1.01 (0.97–1.05)	1.01 (0.97–1.05)
Q4 (highest)	1,559,055	3,758	7,666,154	49.02	0.90 (0.86–0.94)	0.98 (0.94–1.02)	0.97 (0.93–1.01)
**Baseline HDL cholesterol**							
Q1 (lowest)	1,450,022	5,056	7,048,349	71.73	1.00 (reference)	1.00 (reference)	1.00 (reference)
Q2	1,524,625	4,859	7,465,328	65.09	1.03 (0.99–1.08)	1.05 (1.01–1.09)	1.06 (1.02–1.10)
Q3	1,614,880	4,730	7,914,017	59.77	1.03 (0.99–1.08)	1.06 (1.01–1.10)	1.07 (1.03–1.12)
Q4 (highest)	1,540,089	4,158	7,533,444	55.19	1.07 (1.03–1.12)	1.11 (1.06–1.15)	1.13 (1.08–1.18)
**Baseline LDL cholesterol**							
Q1 (lowest)	1,517,736	5,662	7,339,297	77.15	1.00 (reference)	1.00 (reference)	1.00 (reference)
Q2	1,547,973	4,915	7,569,841	64.93	0.96 (0.93–1.00)	1.03 (0.99–1.07)	1.03 (0.99–1.07)
Q3	1,493,483	4,266	7,330,769	58.19	0.93 (0.90–0.97)	1.02 (0.98–1.06)	1.02 (0.98–1.06)
Q4 (highest)	1,570,424	3,960	7,721,230	51.29	0.91 (0.88–0.95)	1.01 (0.96–1.05)	1.00 (0.96–1.04)
**Baseline TG**							
Q1 (lowest)	1,503,719	4,583	7,333,871	62.49	1.00 (reference)	1.00 (reference)	1.00 (reference)
Q2	1,548,052	4,910	7,555,056	64.99	0.97 (0.93–1.01)	0.96 (0.92–1.00)	0.94 (0.91–0.98)
Q3	1,532,448	4,925	7,493,265	65.73	0.97 (0.94–1.01)	0.95 (0.91–0.99)	0.93 (0.89–0.97)
Q4 (highest)	1,545,397	4,385	7,578,945	57.86	0.90 (0.86–0.93)	0.87 (0.83–0.90)	0.84 (0.81–0.88)

*IR* incidence rate, *HR* hazard ratio, *CI* confidence interval, *Q* quartile, *HDL* high-density lipoprotein, *LDL* low-density lipoprotein, *TG* triglycerides.

Model 1: adjusted for age and sex.

Model 2: adjusted for demographic factors (age, sex, income level), and systemic comorbidities (hypertension, diabetes mellitus, stroke, heart disease, chronic kidney disease, and dyslipidemia medication).

Model 3: adjusted for demographic factors, systemic comorbidities, and behavioral factors (smoking history, drinking habits, physical activity, and body mass index).

### Other covariates and exudative age-related macular degeneration

The association between other baseline parameters and the risk of exudative AMD is shown in Supplementary Table 2. Male sex and high income levels were associated with a greater incidence of exudative AMD. All systemic comorbidities included in the study were positively associated with exudative AMD, except for stroke. Smoking status was also associated with an increased risk of exudative AMD.

### Sensitivity analysis

Table 3 demonstrates the results of the sensitivity analyses, excluding those who were taking lipid-lowering medication at baseline. In the fully adjusted analysis (model 3), the highest HDL cholesterol quartile group had a greater risk of exudative AMD than the lowest HDL cholesterol quartile group with an HR (95% CI) of 1.13 (1.07–1.19), and the highest TG quartile group had a lower risk of exudative AMD than the lowest TG quartile group with an HR (95% CI) of 0.82 (0.78–0.87). The results of the sensitivity analyses were similar to those of the main analyses.

**Table 3:** Hazard ratios and 95% confidence intervals for development of exudative age-related macular degeneration according to baseline lipid profile (sensitivity analysis excluding subjects taking lipid-lowering medication at baseline).

	Subject No	Case No	Duration (person-years)	IR per 100,000 person-years	Model 1	Model 2	Model 3
					HR (95% CI)	HR (95% CI)	HR (95% CI)
**Overall**							
**Baseline total cholesterol**							
Q1 (lowest)	1,105,314	3,569	5,356,717	66.63	1.00 (reference)	1.00 (reference)	1.00 (reference)
Q2	1,176,813	3,328	5,773,760	57.64	1.00 (0.96–1.05)	1.01 (0.96–1.06)	1.00 (0.96–1.05)
Q3	1,137,496	2,931	5,593,217	52.40	1.00 (0.95–1.05)	1.00 (0.95–1.05)	0.99 (0.95–1.05)
Q4 (highest)	1,179,743	2,672	5,799,425	46.07	0.98 (0.93–1.03)	0.99 (0.94–1.04)	0.98 (0.93–1.03)
**Baseline HDL cholesterol**							
Q1 (lowest)	1,059,314	3,317	5,162,782	64.25	1.00 (reference)	1.00 (reference)	1.00 (reference)
Q2	1,132,738	3,200	5,556,368	57.59	1.03 (0.98–1.08)	1.04 (0.99–1.09)	1.05 (1.00–1.10)
Q3	1,217,554	3,162	5,976,220	52.91	1.03 (0.98–1.09)	1.05 (1.00–1.10)	1.07 (1.02–1.12)
Q4 (highest)	1,189,760	2,821	5,827,749	48.41	1.08 (1.02–1.13)	1.10 (1.04–1.15)	1.13 (1.07–1.19)
**Baseline LDL cholesterol**							
Q1 (lowest)	1,127,278	3,340	5,477,665	60.97	1.00 (reference)	1.00 (reference)	1.00 (reference)
Q2	1,170,952	3,351	5,736,337	58.42	1.05 (1.00–1.10)	1.05 (1.00–1.10)	1.05 (1.00–1.10)
Q3	1,148,953	3,064	5,645,453	54.27	1.04 (0.99–1.09)	1.04 (0.99–1.10)	1.04 (0.99–1.09)
Q4 (highest)	1,152,183	2,745	5,663,664	48.47	1.03 (0.97–1.08)	1.03 (0.98–1.09)	1.02 (0.97–1.08)
**Baseline TG**							
Q1 (lowest)	1,119,041	3,039	5,468,095	55.58	1.00 (reference)	1.00 (reference)	1.00 (reference)
Q2	1,149,704	3,246	5,621,494	57.74	0.96 (0.92–1.01)	0.96 (0.91–1.01)	0.94 (0.90–0.99)
Q3	1,165,998	3,276	5,711,515	57.36	0.94 (0.90–0.99)	0.93 (0.88–0.97)	0.90 (0.86–0.95)
Q4 (highest)	1,164,623	2,939	5,722,015	51.36	0.87 (0.83–0.91)	0.85 (0.81–0.89)	0.82 (0.78–0.87)

*IR* incidence rate, *HR* hazard ratio, *CI* confidence interval, *Q* quartile, *HDL* high-density lipoprotein, *LDL* low-density lipoprotein, *TG* triglycerides.

Model 1: adjusted for age and sex.

Model 2: adjusted for demographic factors (age, sex, income level), and systemic comorbidities (hypertension, diabetes mellitus, stroke, heart disease, chronic kidney disease, and dyslipidemia medication).

Model 3: adjusted for demographic factors, systemic comorbidities, and behavioral factors (smoking history, drinking habits, physical activity, and body mass index).

## Discussion

This nationwide population-based cohort study revealed the independent influence of lipid profile on the future incidence of exudative AMD in South Korea. A higher baseline HDL cholesterol level and lower baseline TG level were associated with a significantly increased risk of exudative AMD after full adjustment for relevant covariates. This finding was consistently observed in various subgroups of the study population, and the sensitivity analysis that eliminated the influence of anti-dyslipidemia medication on the measured lipid profile also presented very similar results.

Lipid metabolism has long been believed to be involved in AMD pathogenesis. Numerous observational studies have evaluated the association between various serum lipid levels and AMD, but most sample sizes were small and the results were often weak and inconsistent^12–14^. More recently, evidence from large-scale observational studies and genetic analyses consistently indicated that HDL cholesterol level is positively associated with AMD, yet a consensus has not been attained regarding the association between other lipid subfractions (e.g., LDL cholesterol and TG) and AMD. A recent large cross-sectional study of a population of European descent demonstrated that AMD is associated with high HDL cholesterol and low TG levels, while another cohort study revealed that high HDL cholesterol and low LDL cholesterol levels are associated with an increased risk of AMD in populations of European ancestry^22^. Mendelian randomization studies using genetic variants associated with lipid fractions also supported the hypothesis that elevated HDL cholesterol levels are associated with an increased risk of AMD in populations of European descent^15–17^. Regarding other lipid subfractions, however, only one Mendelian randomization study found an inverse correlation between AMD and genetically determined LDL cholesterol/TG levels^17^, while others have found no such association^15,16^. Substantially less epidemiological evidence exists for the association of lipid profile and AMD in Asian populations. A cross-sectional study from South Korea revealed that high HDL is associated with AMD prevalence^3^. Meanwhile, other cohort studies of Asian populations have not revealed any relationship between lipid subfraction and AMD^23,24^, probably due to the small number of cases analyzed. A Mendelian randomization study in an Asian population revealed that AMD is associated with higher genetically determined HDL cholesterol levels, but not with other lipid subfractions. Taken together, conflicting associations between lipid profile and AMD have been reported mainly in the European white populations, and there is growing genetic and epidemiological evidence that high baseline HDL cholesterol levels are associated with a greater risk of AMD.

High HDL cholesterol levels and low TG levels are well-known for their protective effect against cardiovascular diseases and inverse association with obesity^25,26^. Considering that cardiovascular disease and obesity are classic risk factors for AMD^5,6^, it is surprising that high HDL cholesterol and low TG levels are risk factors for AMD. Our study results with multi-model adjustment clearly demonstrate this counterintuitive association. The hazard ratio of the highest HDL cholesterol/TG quartile compared to the lowest quartile was augmented after adjusting for systemic comorbidities (1.07 in model 1 and 1.13 in model 3 for HDL cholesterol; 0.90 in model 1 and 0.84 in model 3 for TG). The opposite direction of association toward AMD compared with cardiovascular disease reflects a more complex involvement of lipid metabolism in the development of AMD. Currently there is no intuitive clinical explanation for the associations found. Studies suggested that most lipids in the retina are synthesized locally and some also reported that most of AMD-associated genes of the lipoprotein metabolism are also expressed locally in the outer retina^12,27^. These suggest a role for retina-specific mechanisms such as local lipid trafficking in AMD pathogenesis.

The complex involvement of lipid metabolism in AMD is also supported by previous genetic studies. Five genetic loci associated with both lipid metabolism and AMD (*ABCA1*, *CETP*, *LIPC*, *APOE*, and *VEGFA*) were identified by previous genome-wide association studies^7,12^. Moreover, Mendelian randomization studies found genetic variants with opposite directions of association between HDL level and AMD. For instance, among the three variants most strongly associated with AMD, one variant in *CETP* loci was associated with both increased HDL cholesterol level and increased AMD risk, while two variants in *LIPC* loci were strongly associated with decreased genetically determined HDL cholesterol level and increased AMD risk^12^. This indicates that not all mechanisms for increasing HDL cholesterol level are involved in increasing the risk of AMD development and careful consideration of the composition, properties, and local metabolism of HDL cholesterol is required.

While the association between HDL cholesterol level and AMD has recently been highlighted by various studies^12,28^, TG level has not been the focus of investigations regarding lipid metabolism in AMD pathogenesis. Our results showed that the baseline TG levels affect the risk of exudative AMD development with an effect size similar to that of HDL cholesterol. This implies that TG-associated metabolism is also involved in AMD pathogenesis. Moreover, the most recent cross-sectional study and Mendelian randomization study showed that genetically predicted TG levels were associated with a decreased risk of different AMD subtypes^17,29^. Thus, future research elucidating the underlying mechanism of an inverse association between TG levels and AMD is warranted.

The strengths of the present study include the large number of Asian subjects of a single ethnicity and the cohort study design. This study provides strong epidemiological evidence for the association between lipid profiles and the future risk of exudative AMD in an Asian population. However, this study has some limitations that need to be recognized. First, we could not include dry AMD as an outcome because it is usually asymptomatic, and only patients who visited the ophthalmology clinic by chance would have been recorded in the claims data, leading to a bias in outcome ascertainment. Second, the study might have missed those who were unable to access the healthcare system. This explains our finding that a higher income level presents a greater risk of exudative AMD. However, we adjusted for income levels in the multivariable analysis, and this would have decreased bias that could have been caused by those who had exudative AMD but did not receive proper treatment. Third, we were unable to measure other confounders, such as dietary factors or niacin supplementation, due to a lack of data. There might be unknown confounding factors that could not be assessed in the present study. Forth, we could not analyze more detailed lipid subfractions as this was not measured in the Korean NHSP. Fifth, we could not differentiate the subtypes of exudative AMD and a large number of polypoidal choroidal vasculopathy (PCV) cases may have been included as cases. However, previous studies on PCV have reported that high HDL is associated with an increased risk of PCV and expected that there is no significant difference in the direction of association with lipid profiles between typical exudative AMD and PCV^16,30^. Lastly, the study participants were those who received health screening, so they might have better health behaviors and more interest in their own health than the general population. However, the Korean National Health Screening is freely provided by the government, and a very large proportion of Korean citizens participate in it (over 70% in both 2013 and 2014)^31^. Therefore, the selection bias would not be large compared to that of other studies.

In conclusion, this nationwide population-based health screening cohort study demonstrated that high baseline HDL cholesterol levels and low baseline TG levels are independent risk factors for exudative AMD development in Koreans. Future studies are needed to elucidate in detail how lipid metabolism is involved in AMD pathogenesis.

## Supplementary Information

Supplementary Information.

## Data Availability

The datasets analyzed in the current study were provided by the Korean NHIS. The data are available at https://nhiss.nhis.or.kr with the permission of the NHIS.

## References

[1] Bourne RR (2013). Causes of vision loss worldwide, 1990–2010: A systematic analysis. Lancet Glob. Health.

[2] Wong WL (2014). Global prevalence of age-related macular degeneration and disease burden projection for 2020 and 2040: A systematic review and meta-analysis. Lancet Glob. Health.

[3] Park SJ (2014). Age-related macular degeneration: prevalence and risk factors from Korean National Health and Nutrition Examination Survey, 2008 through 2011. Ophthalmology.

[4] Lim LS, Mitchell P, Seddon JM, Holz FG, Wong TY (2012). Age-related macular degeneration. Lancet.

[5] Snow KK, Seddon JM (1999). Do age-related macular degeneration and cardiovascular disease share common antecedents?. Ophthalmic Epidemiol..

[6] Chakravarthy U (2010). Clinical risk factors for age-related macular degeneration: A systematic review and meta-analysis. BMC Ophthalmol..

[7] Fritsche LG (2016). A large genome-wide association study of age-related macular degeneration highlights contributions of rare and common variants. Nat. Genet..

[8] Klein RJ (2005). Complement factor H polymorphism in age-related macular degeneration. Science.

[9] Neale BM (2010). Genome-wide association study of advanced age-related macular degeneration identifies a role of the hepatic lipase gene (LIPC). Proc. Natl. Acad. Sci. U. S. A..

[10] Yu Y (2011). Common variants near FRK/COL10A1 and VEGFA are associated with advanced age-related macular degeneration. Hum. Mol. Genet..

[11] Wang L (2010). Abundant lipid and protein components of drusen. PLoS ONE.

[12] van Leeuwen EM (2018). A new perspective on lipid research in age-related macular degeneration. Prog. Retin. Eye Res..

[13] Cheung CMG (2017). Plasma lipoprotein subfraction concentrations are associated with lipid metabolism and age-related macular degeneration. J. Lipid Res..

[14] Kersten E (2018). Systemic and ocular fluid compounds as potential biomarkers in age-related macular degeneration. Surv. Ophthalmol..

[15] Burgess S, Smith GD (2017). Mendelian randomization implicates high-density lipoprotein cholesterol-associated mechanisms in etiology of age-related macular degeneration. Ophthalmology.

[16] Fan Q (2017). HDL-cholesterol levels and risk of age-related macular degeneration: A multiethnic genetic study using Mendelian randomization. Int. J. Epidemiol..

[17] Han X, Ong JS, Hewitt AW, Gharahkhani P, MacGregor S (2021). The effects of eight serum lipid biomarkers on age-related macular degeneration risk: A Mendelian randomization study. Int. J. Epidemiol..

[18] Seong SC (2017). Data resource profile: The national health information database of the national health insurance service in South Korea. Int. J. Epidemiol..

[19] Park SJ, Kwon KE, Choi NK, Park KH, Woo SJ (2015). Prevalence and incidence of exudative age-related macular degeneration in South Korea: A nationwide population-based study. Ophthalmology.

[20] Hwang S (2022). High-density lipoprotein cholesterol and the risk of future retinal artery occlusion development: A nationwide cohort study. Am. J. Ophthalmol..

[21] World Health Organization. Regional Office for the Western P (2000). The Asia-Pacific Perspective: Redefining Obesity and its Treatment.

[22] Nordestgaard LT, Tybjaerg-Hansen A, Frikke-Schmidt R, Nordestgaard BG (2021). Elevated apolipoprotein A1 and HDL cholesterol associated with age-related macular degeneration: 2 population cohorts. J. Clin. Endocrinol. Metab..

[23] Cheung CMG (2017). Six-year incidence of age-related macular degeneration in Asian Malays: The Singapore Malay eye study. Ophthalmology.

[24] Cheung CM (2012). Prevalence of and risk factors for age-related macular degeneration in a multiethnic Asian cohort. Arch. Ophthalmol..

[25] Rader DJ, Hovingh GK (2014). HDL and cardiovascular disease. Lancet.

[26] Nordestgaard BG, Varbo A (2014). Triglycerides and cardiovascular disease. Lancet.

[27] Lin JB (2016). Cholesterol in mouse retina originates primarily from in situ de novo biosynthesis. J. Lipid Res..

[28] Betzler BK, Rim TH, Sabanayagam C, Cheung CMG, Cheng CY (2020). High-density lipoprotein cholesterol in age-related ocular diseases. Biomolecules.

[29] Colijn JM (2019). Increased high-density lipoprotein levels associated with age-related macular degeneration: Evidence from the EYE-RISK and European Eye Epidemiology Consortia. Ophthalmology.

[30] Liu K (2014). Genes in the high-density lipoprotein metabolic pathway in age-related macular degeneration and polypoidal choroidal vasculopathy. Ophthalmology.

[31] KOSIS KOrean Statistical Information Service. http://kosis.kr/eng/index/index.do. Accessed March 14, 2021.

