# Supplementary material for: Lipid profile and future risk of exudative age-related macular degeneration development: a nationwide cohort study from South Korea
Source: Sci Rep. 2022 Nov 5;12:18777. doi: 10.1038/s41598-022-23607-w (PMC9637211; doi:10.1038/s41598-022-23607-w)

Lipid profile and future risk of exudative age-related macular degeneration development: A nationwide cohort study from South Korea

Sungsoon Hwang, MD[1,2]; Se Woong Kang, MD, PhD[1]; Jaehwan Choi, MD[1]; Ki Young Son, MD[1]; Dong Hui Lim, MD, PhD[1,2]; Dong Wook Shin, MD, PhD[2,3,4]; Kyunga Kim, PhD[4,5]; Sang Jin Kim, MD, PhD[1]

[1]Department of Ophthalmology, Samsung Medical Center, Sungkyunkwan University School of Medicine, Seoul, Republic of Korea

[2]Department of Clinical Research Design and Evaluation, Samsung Advanced Institute for Health Sciences and Technology (SAIHST), Sungkyunkwan University, Seoul, Republic of Korea

[3]Department of Family Medicine and Supportive Care Center, Samsung Medical Center, Sungkyunkwan University School of Medicine, Seoul, Republic of Korea

[4]Department of Digital Health, Samsung Advanced Institute for Health Sciences and Technology (SAIHST), Sungkyunkwan University, Seoul, Republic of Korea

[5]Statistics and Data Center, Research Institute for Future Medicine, Samsung Medical Center, Seoul, Republic of Korea.

Correspondence

Sang Jin Kim, MD, PhD

Department of Ophthalmology, Samsung Medical Center, Sungkyunkwan University School of Medicine, #81 Irwon-ro, Gangnam-gu, Seoul 06351, Republic of Korea

Tel: +82-2-3410-3548, Fax: +82-2-3410-0074

E-mail: sangjin.kim.md@gmail.com

Short Title: Lipid profile and exudative AMD

Keywords: age-related macular degeneration, cholesterol, high-density lipoprotein, low-density lipoprotein, triglycerides

Supplemental Table 1. Baseline characteristics of the study population according to development of exudative age-related macular degeneration

Variables	Exudative AMD No (N = 6,110,813)	Exudative AMD Yes (N = 18,803)	p-value
1. Demographic Factors			
Age, years, mean ± SD	60.78 ± 8.42	67.69 ± 8.47	<.001
Age group, No. (%)			<.001
50–59 years	3,145,026 (51.47)	3,532 (18.78)	
60–69 years	1,842,555 (30.15)	6,606 (35.13)	
70–79 years	949,816 (15.54)	7,075 (37.63)	
80–89 years	164,407 (2.69)	1,546 (8.22)	
≥90 years	9,009 (0.15)	44 (0.23)	
Sex, No. (%)			<.001
Male	2,921,789 (47.81)	11,913 (63.36)	
Female	3,189,024 (52.19)	6,890 (36.64)	
Income, No. (%)			<.001
Q1 (lowest)	1,274,965 (20.86)	3,519 (18.72)	
Q2	1,091,081 (17.85)	2,851 (15.16)	
Q3	1,414,037 (23.14)	4,220 (22.44)	
Q4 (highest)	2,330,730 (38.14)	8,213 (43.68)	
2. Systemic Comorbidities			
Hypertension, No. (%)			<.001
No	3,572,837 (58.47)	8,173 (43.47)	
Yes	2,537,976 (41.53)	10,630 (56.53)	
Diabetes mellitus, No. (%)			<.001
No	4,968,821 (81.31)	13,919 (74.03)	
Yes	1,141,992 (18.69)	4,884 (25.97)	
Dyslipidemia medication, No. (%)			<.001
No	4,586,866 (75.06)	12,500 (66.48)	
Yes	1,523,947 (24.94)	6,303 (33.52)	
Stroke, No. (%)			<.001
No	6,005,226 (98.27)	18,311 (97.38)	
Yes	105,587 (1.73)	492 (2.62)	
Heart diseases, No. (%)			<.001
No	5,859,124 (95.88)	17,391 (92.49)	
Yes	251,689 (4.12)	1,412 (7.51)	
Chronic kidney disease, No. (%)			<.001
No	5,690,764 (93.13)	16,444 (87.45)	
Yes	420,049 (6.87)	2,359 (12.55)	
3. Behavioral Factors			
Smoking history, No. (%)			<.001
Never smoked	4,018,442 (65.76)	10,287 (54.71)	
Former smoker	1,122,769 (18.37)	5,263 (27.99)	
Current smoker	969,602 (15.87)	3,253 (17.30)	
Drinking habit, No. (%)			<.001
None	3,908,104 (63.95)	12,097 (64.34)	
Mild	1,914,687 (31.33)	5,437 (28.92)	
Heavy	288,022 (4.71)	1,269 (6.75)	
Regular physical activity, No. (%)			<.001
No	4,677,734 (76.55)	14,030 (74.62)	
Yes	1,433,079 (23.45)	4,773 (25.38)	
Body mass index, No (%)			<.001
< 18.5 kg/m^2	139,806 (2.29)	456 (2.43)	
18.5 to < 23 kg/m^2	2,124,758 (34.77)	6,149 (32.70)	

23 to < 25 kg/m^2	1,651,112 (27.02)	5,318 (28.28)	
25 to < 30 kg/m^2	1,980,165 (32.40)	6,300 (33.51)	
≥ 30 kg/m^2	214,972 (3.52)	580 (3.08)	
4. Examination Results			
Systolic blood pressure, mmHg, mean ± SD	124.57 ± 14.88	126.83 ± 14.81	<.001
Diastolic blood pressure, mmHg, mean ± SD	76.74 ± 9.71	76.50 ± 9.52	<.001
Fasting plasma glucose, mg/dL, mean ± SD	103.32 ± 25.83	105.76 ± 26.39	<.001
Creatinine, mg/dL, mean ± SD	0.90 ± 0.49	0.98 ± 0.63	<.001
Total cholesterol, mg/dL, mean ± SD	197.91 ± 38.56	190.80 ± 38.10	<.001
Total cholesterol group, No. (%)			<.001
Q1 (<172 mg/dL)	1,518,343 (24.85)	5,970 (31.75)	
Q2 (172 to <196 mg/dL)	1,491,635 (24.41)	4,702 (25.01)	
Q3 (196 to <222 mg/dL)	1,545,538 (25.29)	4,373 (23.26)	
Q4 (≥222 mg/dL)	1,555,297 (25.45)	3,758 (19.99)	
HDL cholesterol, mg/dL, mean ± SD	53.83 ± 15.10	52.64 ± 15.48	<.001
HDL cholesterol group, No. (%)			<.001
Q1 (<44 mg/dL)	1,444,966 (23.65)	5,056 (26.89)	
Q2 (44 to <52 mg/dL)	1,519,766 (24.87)	4,859 (25.84)	
Q3 (52 to <62 mg/dL)	1,610,150 (26.35)	4,730 (25.16)	
Q4 (≥62 mg/dL)	1,535,931 (25.13)	4,158 (22.11)	
LDL cholesterol, mg/dL, mean ± SD	118.16 ± 39.25	112.67 ± 35.56	<.001
LDL cholesterol group, No. (%)			<.001
Q1 (<94 mg/dL)	1,512,074 (24.74)	5,662 (30.11)	
Q2 (94 to <117 mg/dL)	1,543,058 (25.25)	4,915 (26.14)	
Q3 (117 to <140 mg/dL)	1,489,217 (24.37)	4,266 (22.69)	
Q4 (≥140 mg/dL)	1,566,464 (25.63)	3,960 (21.06)	
TG, mg/dL, mean ± SD	133.03 ± 87.89	129.67 ± 82.13	<.001
TG group, No. (%)			<.001
Q1 (<79 mg/dL)	1,499,136 (24.53)	4,583 (24.37)	
Q2 (79 to <112 mg/dL)	1,543,142 (25.25)	4,910 (26.11)	
Q3 (112 to <161 mg/dL)	1,527,523 (25.00)	4,925 (26.19)	
Q4 (≥161 mg/dL)	1,541,012 (25.22)	4,385 (23.32)	

AMD, age-related macular degeneration; SD, standard deviation; Q, quartile; HDL, high-density lipoprotein; LDL, low-density lipoprotein ; TG, triglycerides

Supplemental Table 2. Hazard ratios and 95% confidence intervals for the development of exudative AMD according to covariates

	Subject No.	Case No.	Duration (person-years)	IR per 100,000 person-years	Model 1 HR (95% CI)	Model 2 HR (95% CI)	Model 3 HR (95% CI)
1. Demographic Factors							
Age group							
50–59 years	3,148,558	3,532	15,526,701	22.75	1.00 (reference)	1.00 (reference)	1.00 (reference)
60–69 years	1,849,161	6,606	9,040,921	73.07	3.28 (3.15–3.42)	3.14 (3.01–3.27)	3.15 (3.02–3.28)
70–79 years	956,891	7,075	4,620,973	153.11	6.96 (6.68–7.24)	6.35 (6.09–6.62)	6.47 (6.20–6.76)
80–89 years	165,953	1,546	742,503	208.22	9.83 (9.26–10.43)	8.83 (8.30–9.40)	9.15 (8.58–9.75)
≥90 years	9,053	44	30,039	146.48	7.42 (5.51–9.99)	6.79 (5.04–9.15)	7.22 (5.36–9.74)
Sex							
Male	2,933,702	11,913	14,287,883	83.38	1.00 (reference)	1.00 (reference)	1.00 (reference)
Female	3,195,914	6,890	15,673,254	43.96	0.49 (0.48–0.51)	0.49 (0.47–0.50)	0.58 (0.56–0.61)
Income							
Q1 (lowest)	1,278,484	3,519	6,270,529	56.12	1.00 (reference)	1.00 (reference)	1.00 (reference)
Q2	1,093,932	2,851	5,372,521	53.07	0.98 (0.93–1.03)	0.99 (0.94–1.04)	0.99 (0.94–1.04)
Q3	1,418,257	4,220	6,948,394	60.73	1.00 (0.96–1.05)	1.01 (0.96–1.05)	1.01 (0.96–1.05)
Q4 (highest)	2,338,943	8,213	11,369,693	72.24	1.09 (1.05–1.13)	1.09 (1.04–1.13)	1.09 (1.05–1.13)
2. Systemic Comorbidities							
Hypertension							
No	3,581,010	8,173	17,575,211	46.50	1.00 (reference)	1.00 (reference)	1.00 (reference)
Yes	2,548,606	10,630	12,385,926	85.82	1.18 (1.14–1.21)	1.10 (1.07–1.14)	1.10 (1.07–1.14)
Diabetes mellitus							
No	4,982,740	13,919	24,431,099	56.97	1.00 (reference)	1.00 (reference)	1.00 (reference)
Yes	1,146,876	4,884	5,530,038	88.32	1.15 (1.11–1.18)	1.06 (1.02–1.09)	1.05 (1.01–1.09)
Dyslipidemia medication							
No	4,599,366	12,500	22,523,119	55.50	1.00 (reference)	1.00 (reference)	1.00 (reference)
Yes	1,530,250	6,303	7,438,018	84.74	1.25 (1.21–1.29)	1.18 (1.14–1.22)	1.16 (1.13–1.20)
Stroke							
No	6,023,537	18,311	29,460,059	62.16	1.00 (reference)	1.00 (reference)	1.00 (reference)
Yes	106,079	492	501,078	98.19	0.97 (0.88–1.06)	0.89 (0.81–0.97)	0.88 (0.80–0.96)
Heart diseases							
No	5,876,515	17,391	28,749,707	60.49	1.00 (reference)	1.00 (reference)	1.00 (reference)
Yes	253,101	1,412	1,211,430	116.56	1.20 (1.14–1.27)	1.07 (1.02–1.14)	1.06 (1.00–1.12)
Chronic kidney disease							
No	5,707,208	16,444	27,946,077	58.84	1.00 (reference)	1.00 (reference)	1.00 (reference)

Yes	422,408	2,359	2,015,060	117.07	1.18 (1.13–1.23)	1.12 (1.07–1.18)	1.12 (1.07–1.17)
3. Behavioral Factors							
Smoking history							
Never smoked	4,028,729	10,287	19,721,829	52.16	1.00 (reference)	1.00 (reference)	1.00 (reference)
Former smoker	1,128,032	5,263	5,501,240	95.67	1.39 (1.34–1.45)	1.37 (1.32–1.43)	1.38 (1.33–1.44)
Current smoker	972,855	3,253	4,738,068	68.66	1.32 (1.26–1.38)	1.34 (1.28–1.40)	1.36 (1.30–1.43)
Drinking habit							
None	3,920,201	12,097	19,125,031	63.25	1.00 (reference)	1.00 (reference)	1.00 (reference)
Mild	1,920,124	5,437	9,430,566	57.65	0.97 (0.93–1.00)	0.97 (0.94–1.01)	0.92 (0.89–0.96)
Heavy	289,291	1,269	1,405,540	90.29	1.03 (0.97–1.10)	1.05 (0.99–1.12)	0.99 (0.93–1.06)
Regular physical activity							
No	4,691,764	14,030	22,903,993	61.26	1.00 (reference)	1.00 (reference)	1.00 (reference)
Yes	1,437,852	4,773	7,057,144	67.63	1.10 (1.06–1.14)	1.09 (1.06–1.13)	1.09 (1.06–1.13)
Body mass index							
<18.5 kg/m^2	140,262	456	651,484	69.99	0.94 (0.85–1.03)	0.97 (0.88–1.07)	0.96 (0.88–1.06)
18.5 to <23 kg/m^2	2,130,907	6,149	10,390,547	59.18	1.00 (reference)	1.00 (reference)	1.00 (reference)
23 to <25 kg/m^2	1,656,430	5,318	8,126,872	65.44	1.09 (1.05–1.13)	1.06 (1.02–1.10)	1.06 (1.02–1.10)
25 to <30 kg/m^2	1,986,465	6,300	9,740,958	64.68	1.08 (1.04–1.12)	1.03 (0.99–1.06)	1.03 (1.00–1.07)
≥30 kg/m^2	215,552	580	1,051,277	55.17	1.06 (0.97–1.15)	0.97 (0.89–1.06)	0.98 (0.90–1.07)

IR, incidence rate; HR, hazard ratio; CI, confidence interval; Q, quartile.

Model 1: adjusted for age and sex

Model 2: adjusted for demographic factors (age, sex, income level), and systemic comorbidities (hypertension, diabetes mellitus, stroke, heart disease, chronic kidney disease, and dyslipidemia medication)

Model 3: adjusted for demographic factors, systemic comorbidities, and behavioral factors (smoking history, drinking habits, physical activity, and body mass index)

Supplemental Figure 1. Prospective association of total cholesterol with exudative age-related macular degeneration incidence in various subgroups. The risk of exudative age-related macular degeneration in the lowest quartile (Q1, reference group) versus the highest quartile (Q4) was estimated using the Cox proportional hazards model fully adjusted for demographic factors, systemic comorbidities, and behavioral factors (model 3). No significant interactions were observed, and the associations were consistent in the various subgroups.

Total cholesterol

Subgroups		HR (95% CI)	*P* interaction
Age	<70 years	0.97 (0.91–1.02)	0.70
	≥70 years	0.98 (0.91–1.04)	
Sex	Male	0.97 (0.91–1.02)	0.74
	Female	0.97 (0.91–1.04)	
Income level	Q1	1.05 (0.95–1.16)	0.15
	Q2-4	0.95 (0.90–1.01)	
Dyslipidemia medication	No	0.96 (0.90–1.03)	0.49
	Yes	0.99 (0.93–1.05)	
Hypertension	No	0.97 (0.92–1.02)	0.31
	Yes	0.99 (0.90–1.08)	
Diabetes mellitus	No	0.97 (0.92–1.02)	0.63
	Yes	0.97 (0.89–1.05)	
Stroke	No	0.97 (0.93–1.02)	0.31
	Yes	0.81 (0.59–1.11)	
Heart diseases	No	0.97 (0.93–1.02)	0.90
	Yes	0.91 (0.74–1.12)	
Chronic kidney disease	No	0.96 (0.92–1.01)	0.24
	Yes	1.05 (0.93–1.18)	
Smoking history	Never	0.99 (0.93–1.04)	0.88
	Past/Current	0.95 (0.89–1.01)	
Drinking habit	None	1.00 (0.94–1.05)	0.11
	Mild/Heavy	0.92 (0.84–1.01)	
Regular exercise	No	1.01 (0.96–1.06)	0.10
	Yes	0.92 (0.83–1.02)	
Obesity (BMI≥25kg/m^2)	No	0.97 (0.92–1.03)	0.47
	Yes	0.96 (0.89–1.03)	

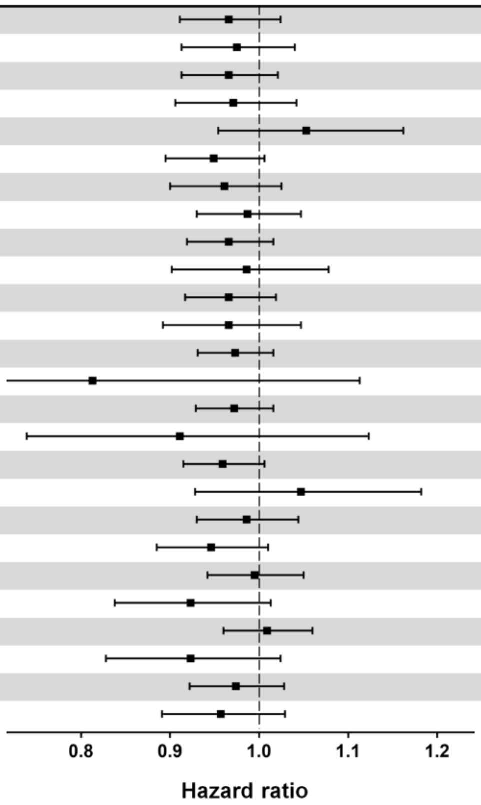

Supplemental Figure 2. Prospective association of high-density lipoprotein cholesterol with incident exudative age-related macular degeneration in various subgroups. The risk of exudative age-related macular degeneration in the lowest quartile (Q1, reference group) versus the highest quartile (Q4) was estimated using the Cox proportional hazards model fully adjusted for demographic factors, systemic comorbidities, and behavioral factors (model 3). No significant interactions were observed, and the associations were consistent in the various subgroups.

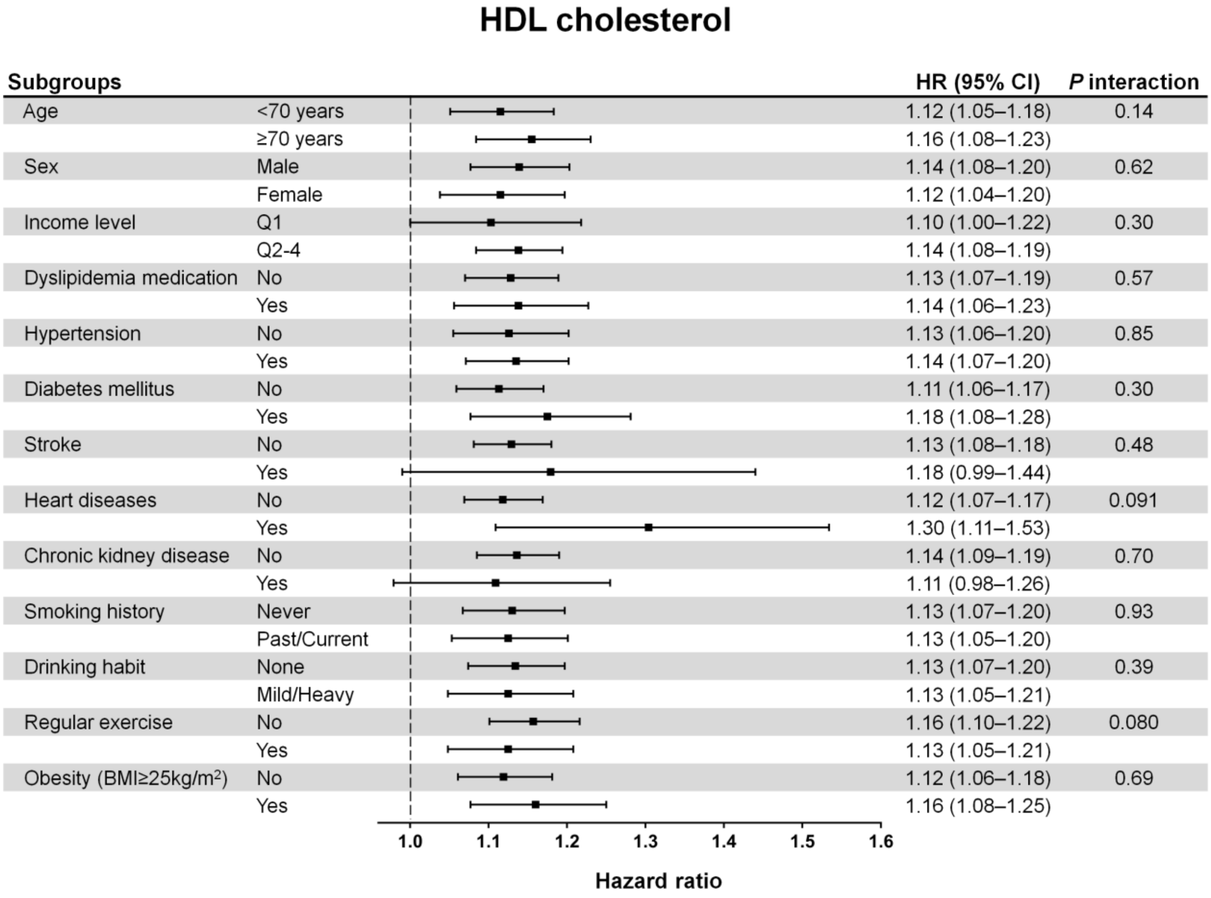

Supplemental Figure 3. Prospective association of low-density lipoprotein cholesterol with incident exudative age-related macular degeneration in various subgroups. The risk of exudative age-related macular degeneration in the lowest quartile (Q1, reference group) versus the highest quartile (Q4) was estimated using the Cox proportional hazards model fully adjusted for demographic factors, systemic comorbidities, and behavioral factors (model 3). No significant interactions were observed.

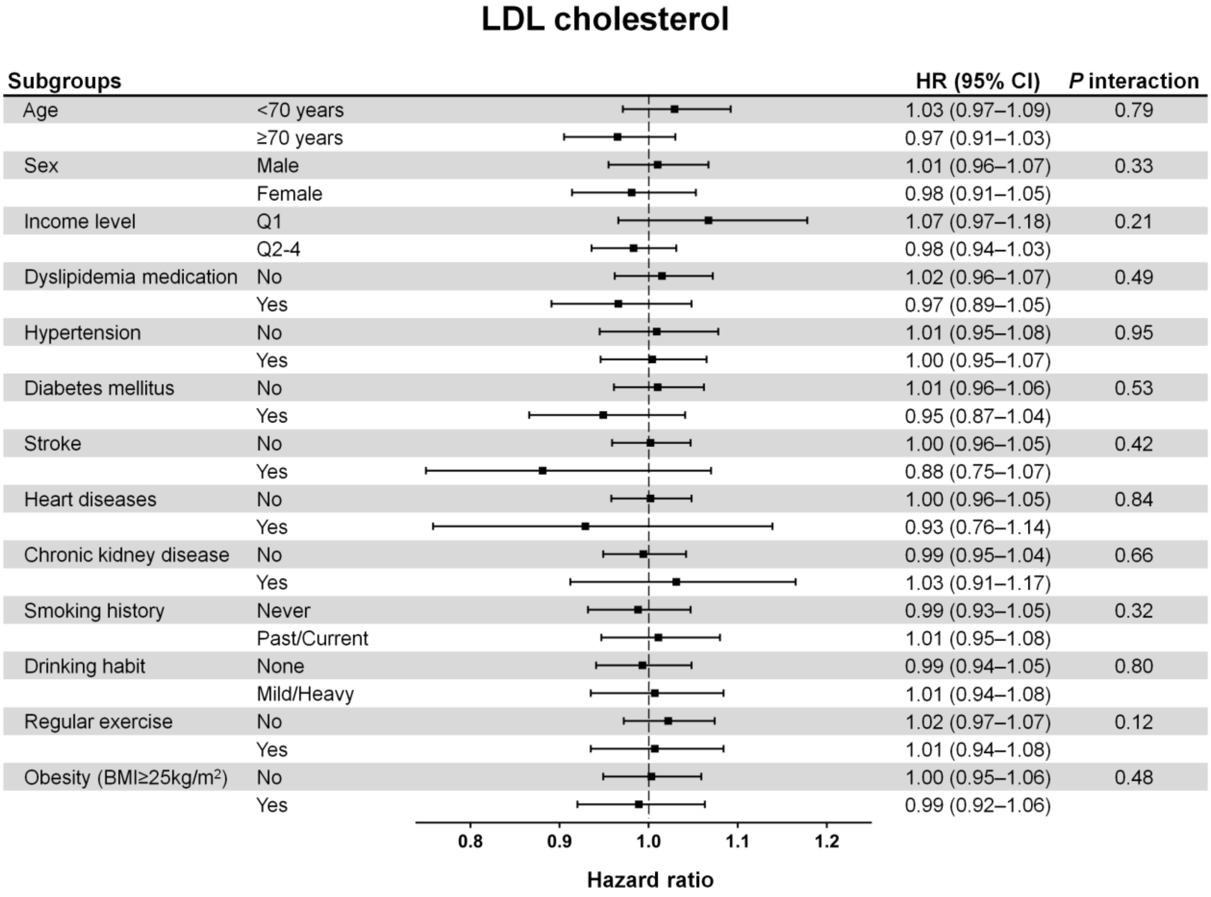

Supplemental Figure 4. Prospective association of triglycerides with incident exudative age-related macular degeneration in various subgroups. The risk of exudative age-related macular degeneration in the lowest quartile (Q1, reference group) versus the highest quartile (Q4) was estimated using the Cox proportional hazards model fully adjusted for demographic factors, systemic comorbidities, and behavioral factors (model 3). No significant interactions were observed, and the associations were consistent in the various subgroups.

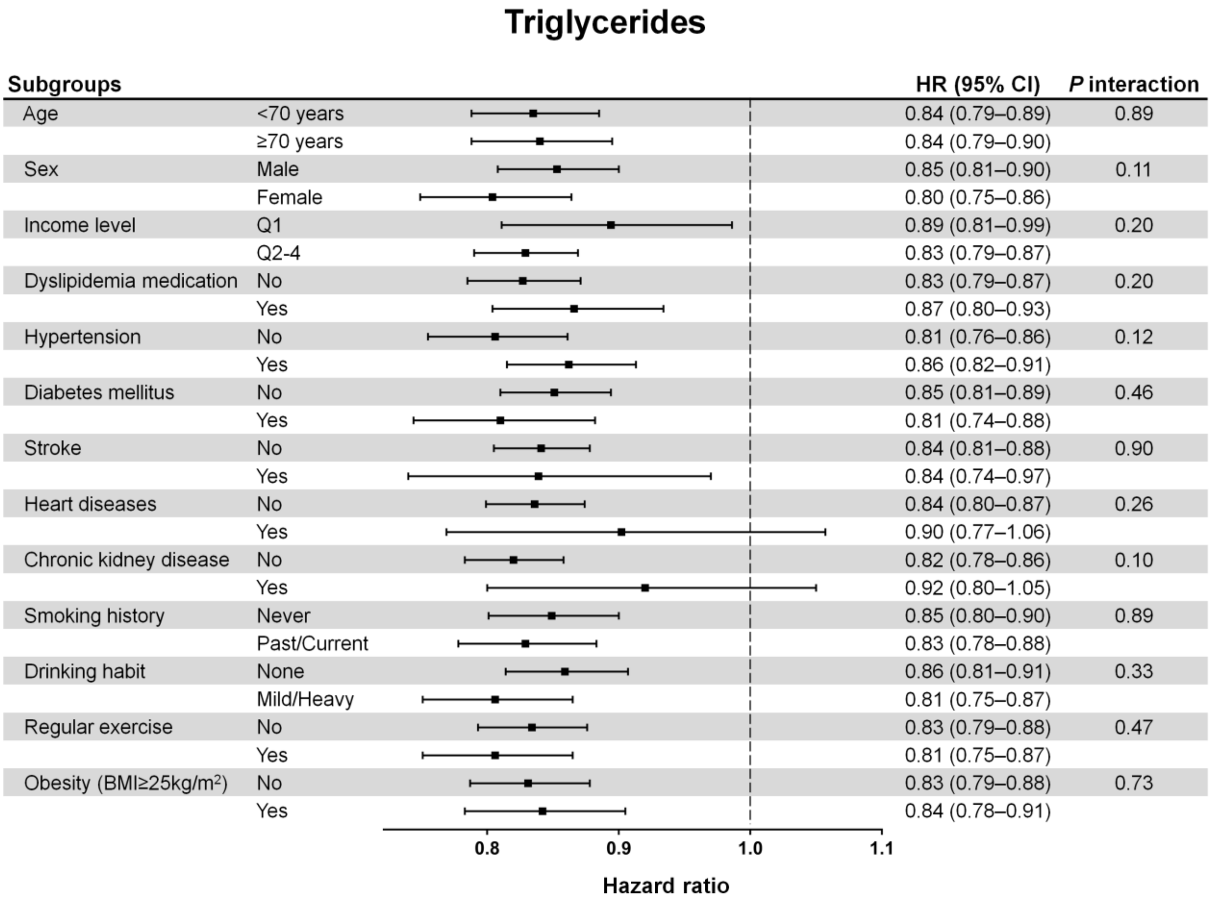

Supplement: Supplementary file 1 — Supplementary Information. [file 41598_2022_23607_MOESM1_ESM.pdf]
